# Apoptosis-Related Signature Predicts Prognosis and Immune Microenvironment Infiltration in Lung Adenocarcinoma

**DOI:** 10.3389/fgene.2022.818403

**Published:** 2022-04-27

**Authors:** Xiaoli Zou, Rong He, Zhenzhen Zhang, Yulan Yan

**Affiliations:** ^1^ Departments of Respiratory Medicine, Affiliated People’s Hospital of Jiangsu University, Zhenjiang, China; ^2^ Departments of Cancer Institute, Affiliated People’s Hospital of Jiangsu University, Zhenjiang, China

**Keywords:** gene signature, lung adenocarcinoma, apoptosis, tumormicroenvironment, immunotherapy

## Abstract

Lung adenocarcinoma (LUAD), a malignancy with high incidence and mortality rates worldwide, contains multiple genomic and epigenomic abnormalities. And the useful tumor markers associated with these abnormalities need further investigation. Whereas apoptosis is a form of programmed cell death, the expression of apoptosis-related genes in LUAD and its relationship with prognosis is unclear. In the present study, we identified 64 differentially expressed apoptosis-related genes (DEARGs) that were differentially expressed between LUAD tissue and normal lung tissue. Based on these DEARGs, all LUAD cases were classified into two subtypes using The Cancer Genome Atlas (TCGA) cohort to assess the prognostic value of apoptosis-related genes for survival. An 11-gene signature was established by applying the Least Absolute Shrinkage and Selection Operator (LASSO) Cox regression method to construct a multigene prediction model and classify all LUAD patients in the TCGA cohort into high or low AS-score groups. Patients in the low AS-score group had significantly higher survival and prognosis than those in the high AS-score group. Taking the median risk score of the AS-score, LUAD patients in the GSE68465 cohort were divided into two risk groups, low and high. The overall survival (OS) time was longer in the low AS-score group. Combined with clinical characteristics, the AS-score was an independent predictor of LUAD patients. Gene ontology (GO) and Kyoto Encylopedia of Genes and Genomes (KEGG) analyses showed that the differential genes between the two groups were mainly enriched in cellular immunity. Further analysis revealed higher immune checkpoint protein expression and higher tumor mutational burden (TMB) in the high AS-score group, suggesting better efficacy of immunotherapy in the high AS-score group than the low AS-score group. And the high AS-score group was better in chemotherapy and targeted therapy efficiency. In conclusion, the AS-score constructed based on apoptosis-related genes can predict the prognosis of LUAD patients and provide some guidance for the antitumor treatment of LUAD patients.

## Introduction

The new Global Cancer Statistics 2020 showed that cancer incidence in China is the highest globally and that lung cancer (LC) remains the second most prevalent malignancy with high mortality (Sung et al., 2021). According to the pathological type of LC, it can be divided into small cell lung cancer (SCLC) and non-small cell lung cancer (NSCLC), of which non-small cell lung cancer includes LUAD and lung squamous carcinoma (LUSC). Meanwhile, LUAD accounts for 85% of non-small cell lung cancer and 40% of all types and is the most common type of LC (Denisenko et al., 2018; Liu L.-P. et al., 2021). With no symptoms in the early stages of LC, it is usually detected at an advanced stage that is not amenable to surgical treatment and has a poor prognosis. The current therapeutic modalities for LUAD include chemotherapy, radiotherapy, targeted drug therapy, immunotherapy, and surgery. However, the sensitivity and specificity of treatment are low due to the heterogeneity of the tumor and the complex immune microenvironment of cancer. Although an increasing number of studies have focused on the analysis of the characteristic death features of tumor cells to predict their prognosis (Liu L.-P. et al., 2021). However, the analysis of molecular features of tumor cell apoptosis to predict lung adenocarcinoma prognosis has not been demonstrated. In this study, we analyzed the molecular features related to tumor apoptosis. The study was carried out to compare the survival differences between the two groups and the efficacy of antitumor drugs by constructing an apoptosis-related prediction model for staging lung adenocarcinoma patients. These suggest that it is crucial to improve treatment specificity and establish a specific prognostic model (Hirsch et al., 2017; Greenawalt et al., 2019).

It is known that there are two main types of cell death, one is programmed death that is finely regulated by the cell, and the other is uncontrolled cell necrosis (Xu et al., 2019). In contrast, apoptosis, as a programming mechanism of cell death, is characterized by specific changes in cell structure and the biochemical processes of all enzyme-catalyzed reactions, mainly removing some damaged and potentially harmful cells from the body (Yaacoub et al., 2016; Carneiro and El-Deiry, 2020). The expression of phosphatidylserine in the outer layer of the cell membrane leads to early recognition and phagocytosis of dead cells by macrophages during apoptosis, without releasing pro-inflammatory cellular components (Hengartner, 2001). Apoptosis is characterized by several morphological features, including membrane vesicles, changes in organelle ultrastructure, and loss of membrane integrity, followed by the emergence of phagocytes that consume the apoptotic cells (Kroemer et al., 2005). The BCL-2 family of proteins is the main apoptosis regulator that directly controls the permeability of membranes (Singh et al., 2019). Cytochrome C is released from mitochondria to form apoptotic vesicles. At the same time, the caspases (cysteine, aspartate-specific proteases) family of proteases plays a crucial executive role in apoptosis (Li and Yuan, 2008), activating the executioner caspase 3 to initiate apoptosis (Ledgerwood and Morison, 2009; Xie et al., 2020). Necrosis, on the other hand, is not regulated and is primarily due to external factors that cause collapse and necrosis, releasing large amounts of harmful substances and causing severe damage to the cellular environment (Degterev et al., 2008).

There is increasing evidence that dysregulation of apoptosis signals the development and progression of malignant tumors, which can become resistant to therapeutic agents due to resistance to apoptosis while evading the immune system (Hassan et al., 2014). The majority of drugs currently used in clinical practice achieve their antitumor effects by affecting the apoptotic signaling pathway (Plati et al., 2008; Giménez-Bonafé et al., 2009).

Current evidence suggests that apoptosis not only plays a role in tumorigenesis, cancer metastasis, cancer immunity, and cancer subtypes, but that senescent or lost apoptotic cells are recognized and phagocytosed by macrophages, leading to the release of cytokines that participate in the complex tumor immune microenvironment, which also influences apoptosis (Carneiro and El-Deiry, 2020).

Apoptosis plays an important role in tumor development and antitumor therapy. However, less study of its specific functions and studies in LUAD drug resistance and tumor immune microenvironment are studied. Therefore, we conducted a systematic analysis to explore the prognostic value of apoptosis-related genes in LUAD and investigate the relevance of apoptosis in LUAD to antitumor drugs and the immune microenvironment.

## Materials and Methods

### LUAD Data Sets and Preprocessing

**FIGURE 1:**
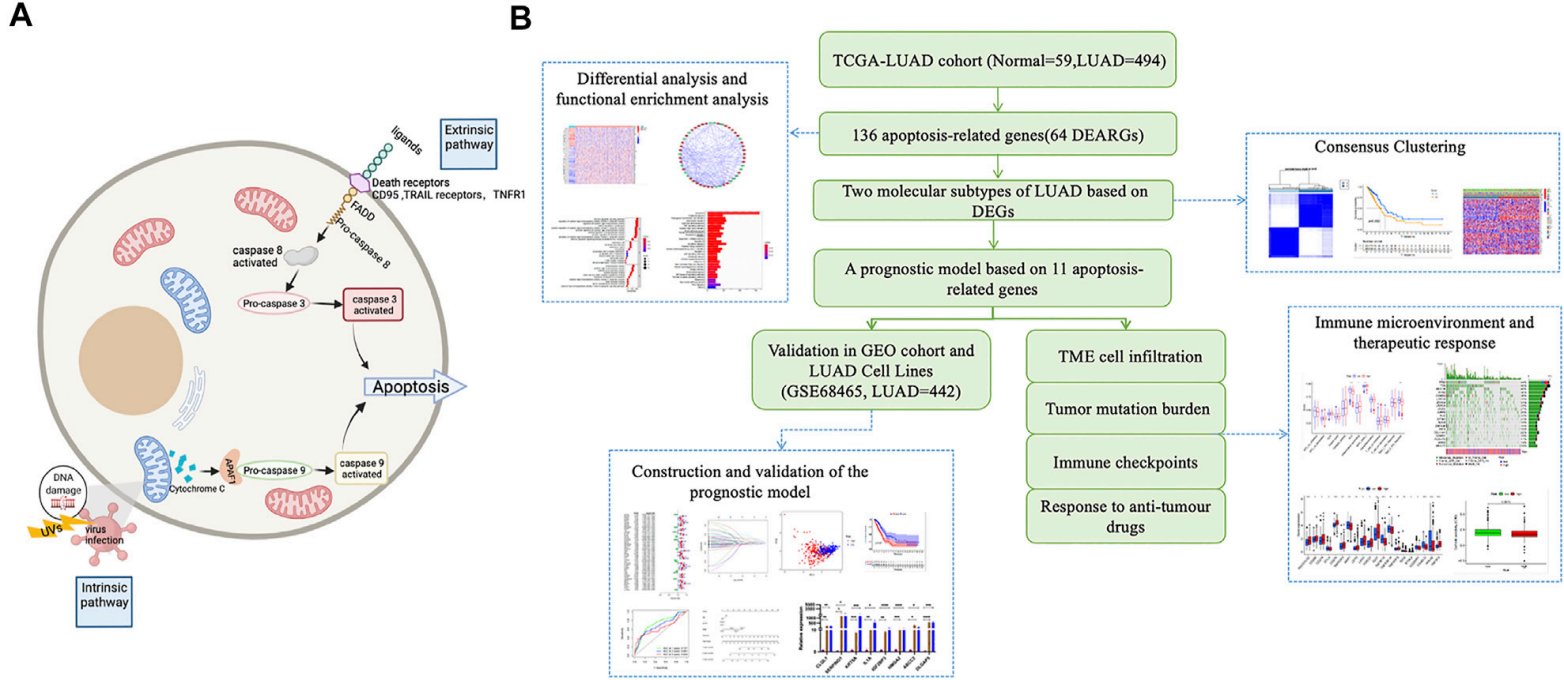
Schematic diagram of the apoptosis pathway and a sketch of the research process. **(A)** Schematic diagram of the apoptosis pathway. **(B)** A sketch of this research process.

Firstly, we draw a simple schematic diagram of the proposed apoptotic process based on the apoptotic pathway (Created with BioRender.com) (Figure 1A). The Cancer Genome Atlas downloaded and opened the LUAD gene expression dataset with complete clinical information, somatic mutation data, and FPKM transcriptome data (TCGA https://portal.gdc.cance r. gov/), excluding samples without survival information, including 494 cases of LUAD and 59 normal tissues. Detailed information on these LUAD patients is presented (Supplementary Table S1). In the KEGG pathway database (https://www.genome.jp/kegg/pathway.html), we found 136 apoptosis-related genes according to the apoptosis pathway (map04210). As no complete set of apoptosis-related genes has been reported before, we searched the literature related to apoptosis, performed a comprehensive analysis, and found that all of them could be found in the set of apoptosis-related genes in the kegg database **(** Supplementary Table S2). GSE68465 (N = 442) was downloaded from the gene expression omnibus (GEO, https://www.ncbi.nlm.nih.gov/geo/) database and was used as the validation set. All data are publicly available online. This study did not require an author to perform experiments on humans or animals. A working diagram showing the overall research process (Figure 1B).

### Data Processing of Differentially Expressed Genes and Functional Enrichment Analyses

The “limma” package was used to identify apoptosis-related genes that were differentially expressed between LUAD and normal tissues in the TCGA database. The screening criteria were A false discovery rate (FDR) < 0.05, |logFC| > 0.5 (Wu et al., 2021). GO and KEGG analysis was performed using the “cluster profile” package based on these differentially expressed genes. The Search Tool for the Retrieval of Interacting Genes (STRING) database (https://st ring-db. org/) was used to input differential genes for PPI information analysis, and Cytoscape software was used to visualize the PPI network.

### Consensus Clustering

Consistent clustering identifies apoptosis-related patterns associated with the expression of apoptosis regulators by the k-means method. The number of clusters and their stability is determined by a consensus clustering algorithm using the “ConsensuClusterPlus” package, repeated 1,000 times to ensure classification stability. The prompt function was used to perform principal component analysis. Heat maps and Kaplan-Meyer (KM) curves were plotted using the R packages “heatmap,” “survminer,” and “survival.”

### Construction and Validation of Apoptosis-Related Gene Signature

The consensus clustering algorithm classifies lung cancer patients into two subtypes, and we next use the R package “limma” to identify differentially expressed genes between subtypes (| logFC | > 2 and FDR <0.001). Univariate Cox regression analysis was used to screen for prognosis-related DEGs, and LASSO—Cox analysis was used to narrow down candidate genes further, resulting in a validated predictive model (Liu et al., 2021b). AS-score = Σ (βi × expi) = 1 (where βi is the coefficient index and gene expression, respectively). The median cut-off value was determined using the “survminer” package. The Kaplan-Meier survival curves were used to identify the time to overall survival (OS) that distinguishes different subtypes of patients. The time-dependent subject operating characteristic curves (ROC) were used to assess the validity and accuracy of the model. The GSE68465 cohort was used as an external validation set to assess the value of the predictive model further.

### TME Cell Infiltration and the Response to Anti-Tumour Drugs

The ssGSEA was performed by the “gsva” software package to calculate the infiltration score of 16 immune cells and the activity of 13 immune-related pathways. The data of the Genomics Of Drug Sensitivity in Cancer (GDSC) platform were used to predict the sensitivity of LUAD patients to common chemotherapeutic drugs and targeted therapeutic drugs (such as cisplatin, paclitaxel, gefitinib, and erlotinib). The “pRRophetic” R package was used to estimate the half-maximal inhibitory concentration (IC50) (Liu et al., 2021c).

### Cell Culture

The human LUAD cell lines (A549 and PC9) and the normal human lung epithelial cell line (BEAS-2B) used in this study were provided by the Institute of Cell Research, Chinese Academy of Sciences (Shanghai, China). The medium for A549 cells was DMEM medium with 10% fetal bovine serum and 1% double antibodies; PC9 and BEAS-2B cells were 1,640 medium with 10% fetal bovine serum and 1% dual antibodies. The cells were placed in a constant temperature incubator with a CO2 concentration of 5% and a temperature of 37°C.

### RNA Extraction and Real-Time PCR

Total RNA was extracted by Trizol reagent (Invitrogen, Carlsbad, CA, United States) according to the instructions. The concentration of the extracted RNA was controlled to be 500 ng/ml with a purity between 1.80 and 2.00. Subsequently, extracted RNA was transcribed using the PrimeScript RT reagent Kit with gDNA Eraser (Takara, Japan). SYBR Green-based real-time PCR was used for analysis. PCR primers were designed and synthesized by Shanghai Bioengineering Co. (Shanghai.China). Primers used for real-time PCR assays are listed in Supplementary Table S3.

### Statistical Analysis

Differences between groups were analyzed using the Wilcoxon test. Independent prognostic analysis was performed using univariate and multivariable cox regression analysis. Correlation tests were performed using Spearman analysis. Survival curves were plotted using log-rank and Kaplan-Meier tests. Mutations between groups were analyzed using the “maftools” R package. *p* < 0.05 was considered statistically significant. Data were processed using R 4.0.5 software.

## Results

### LUAD Dataset Sourcing and Pre-Processing

**FIGURE 2:**
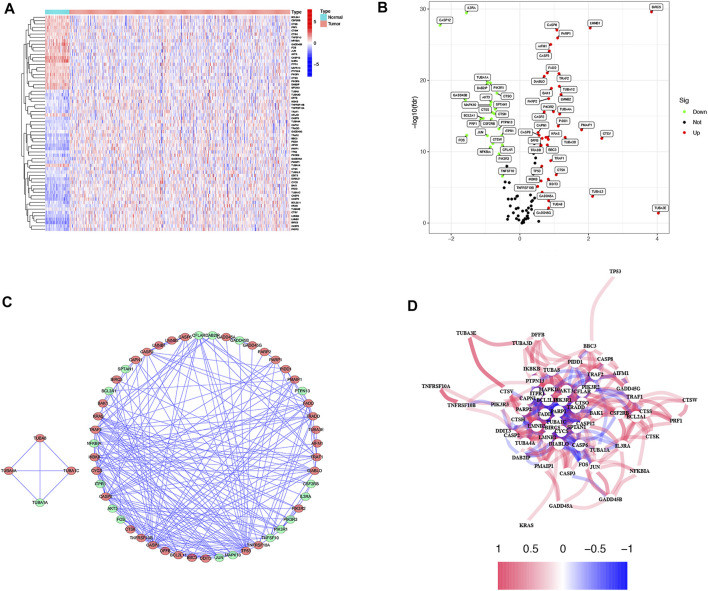
Expressions of the 64 apoptosis-related genes and the interactions among them. **(A)** Heatmap (blue: low expression level; red: high expression level) of the apoptosis-related genes between the normal (N, brilliant blue) and the tumor tissues (T, red). **(B)** The volcano plot shows differential expression of apoptosis genes in LUAD and normal tissue in the TCGA cohort (green: low expression in LUAD; red: high expression in LUAD). **(C)** PPI network shows the apoptosis-related gene interactions (interaction score = 0.7) (green: low expression in LUAD; red: high expression in LUAD). **(D)** The correlation network of the apoptosis-related genes (red line: positive correlation; blue line: negative correlation. The depth of the colors reflects the strength of the relevance).

The TCGA-LUAD database was downloaded from The Cancer Genome Atlas TCGA public database for a total of 594 samples, and patients with no survival information were excluded from further analysis. Comparing 59 normal tissues with 494 LUAD tissues for DEARGs, a total of 64 differentially expressed genes associated with apoptosis were identified, and the heatmap demonstrated the expression of each differential gene in each sample (Figure 2A). Twenty-one of these 64 DEARGs were genetically down-regulated and 38 differentially genetically up-regulated (Figure 2B; Supplementary Table S4). To further explore the interactions between these apoptosis-related genes, we constructed a protein-protein interaction network (PPI) with a minimum required score of 0.7 for PPI analysis, which was used to explore the interconnections between the genes (Figure 2C). Simultaneous drawing of a correlation network containing all apoptosis-related genes (red represents positive correlations and blue represents negative correlations) (Figure 2D).

### Functional Analysis Based on DEARGs

**FIGURE 3:**
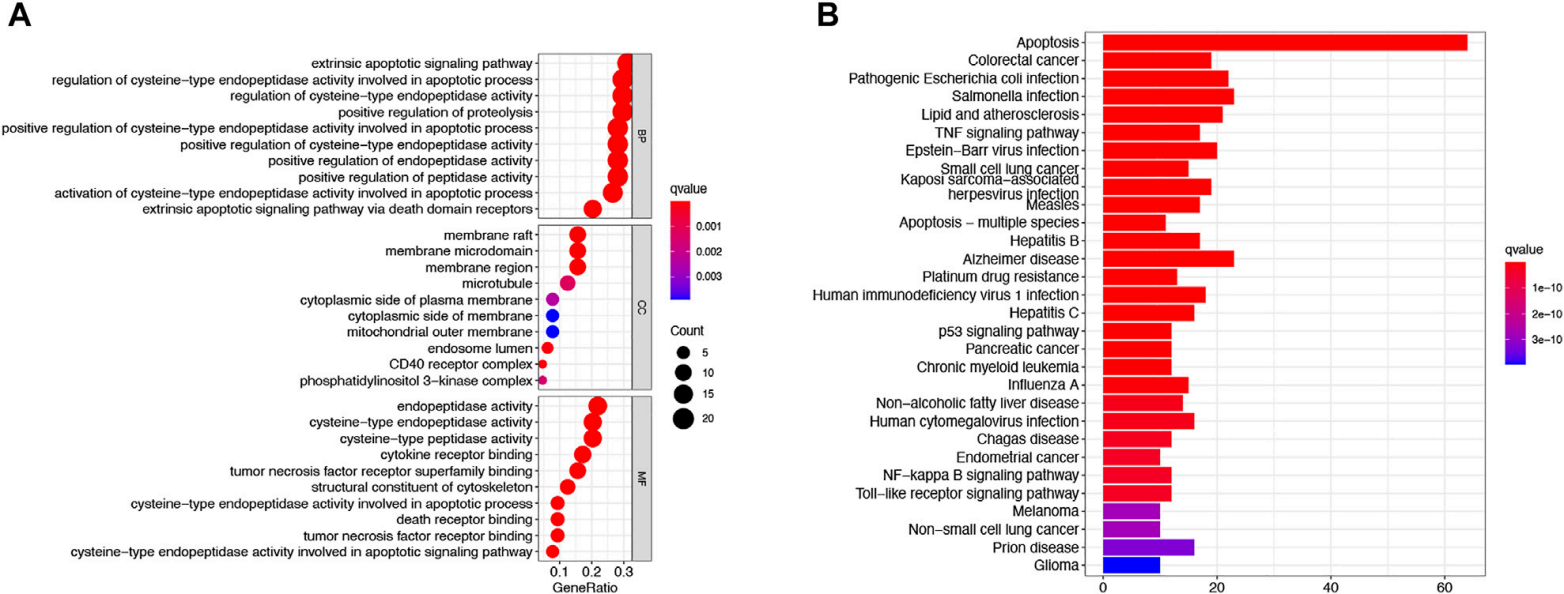
Based on functional analysis of DEARGs between the normal and LUAD groups in the TCGA cohort.**(A)** Bubble shows KEGG pathway analysis predicted DEARGs. The circle size represents the number of genes enriched in the entry, and the color indicates the significance of the *p*-value. **(B)** Barplot shows GO enrichment analysis predicted DEARGs. The color indicates the significance of the *p*-value.

To further explore the differences in the biological, behavioral functions of these DEARGs.We performed GO and KEGG enrichment analyses. The results showed that these 64 DEARGs are mainly involved in apoptosis, *salmonella* infection, and apoptotic pathways and are associated with cysteine-type endopeptidase activity, membrane rafts, and peptide chain endonuclease activity involved in the apoptotic process (Figures 3A,B).

### Molecular Subtypes of LUAD Based on DEGs

**FIGURE 4:**
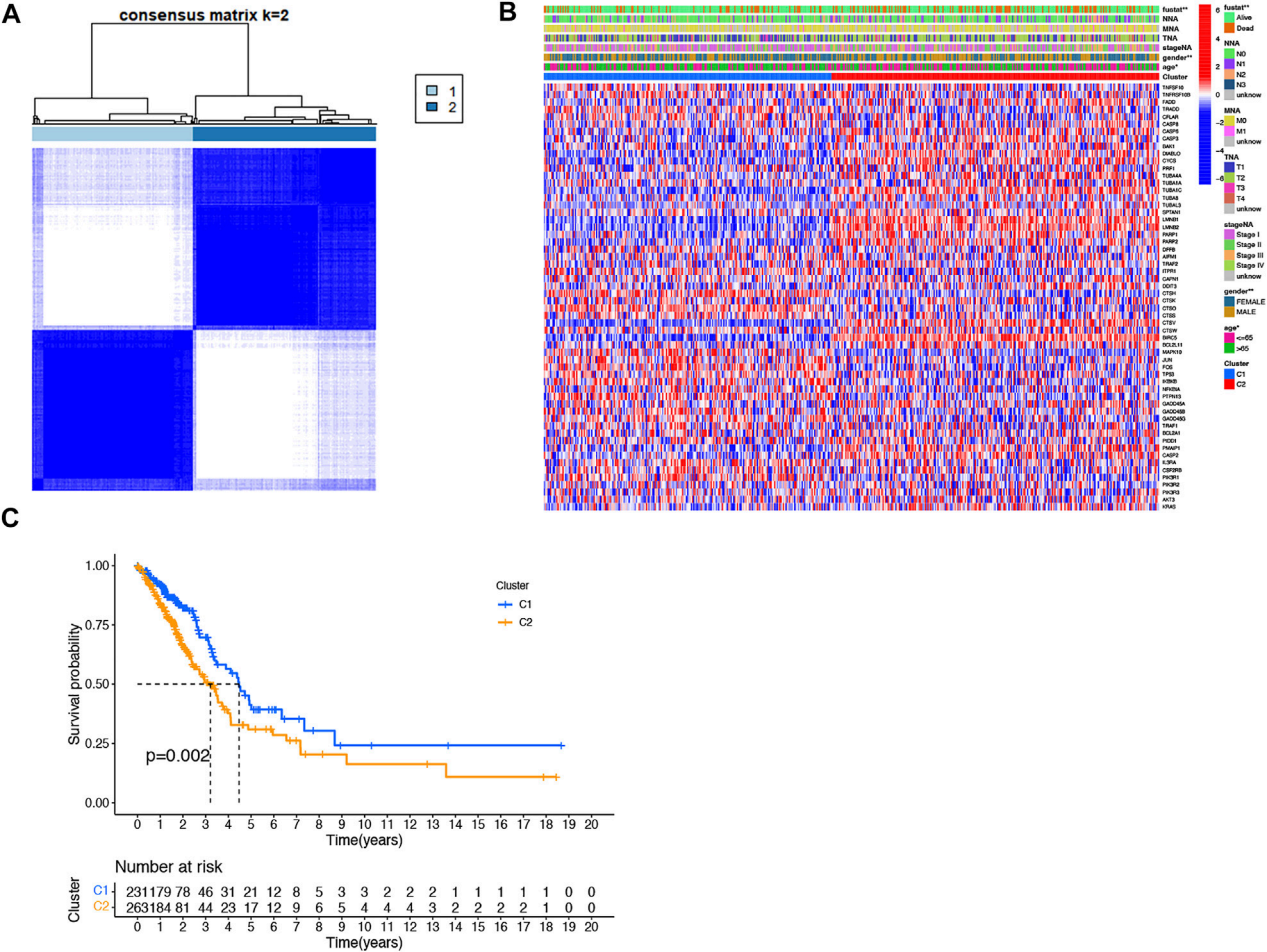
Tumor classification based on the DEARGs. **(A)** 494 LUAD patients were grouped into two clusters according to the consensus clustering matrix (k = 2). **(B)** The heat map shows the clinicopathological characteristics of the differentially expressed genes between the two clusters. **(C)** Kaplan–Meier OS curves for the two clusters.

To explore the association between DEARGs and LUAD, we performed a consensus clustering analysis on all 494 patients with survival information for LUAD in the TCGA cohort. Increasing the clustering variable k from 2 to 9 found that the highest intra-group correlations and lower inter-group correlations when k = 2, indicating that the 494 LUAD patients could be well classified into two clusters based on 64 differentially expressed genes (DEGs) (Figure 4A **)**. The heatmap showed the differential gene expression profile regarding clinical characteristics, including tumor grade, age (≤60 or >60 years), gender, TMN stage, and survival status (alive or dead). The results showed that the C1 group had better clinical performance than the C2 group (Figure 4B). We also compared the overall survival time (OS) of the two groups. There was a significant difference between the two groups (*p* = 0.002). The results suggested that the C1 group has a higher survival rate than the C1 group (Figure 4C).

### Prognostic Gene Modeling in the TCGA Cohort

**FIGURE 5:**
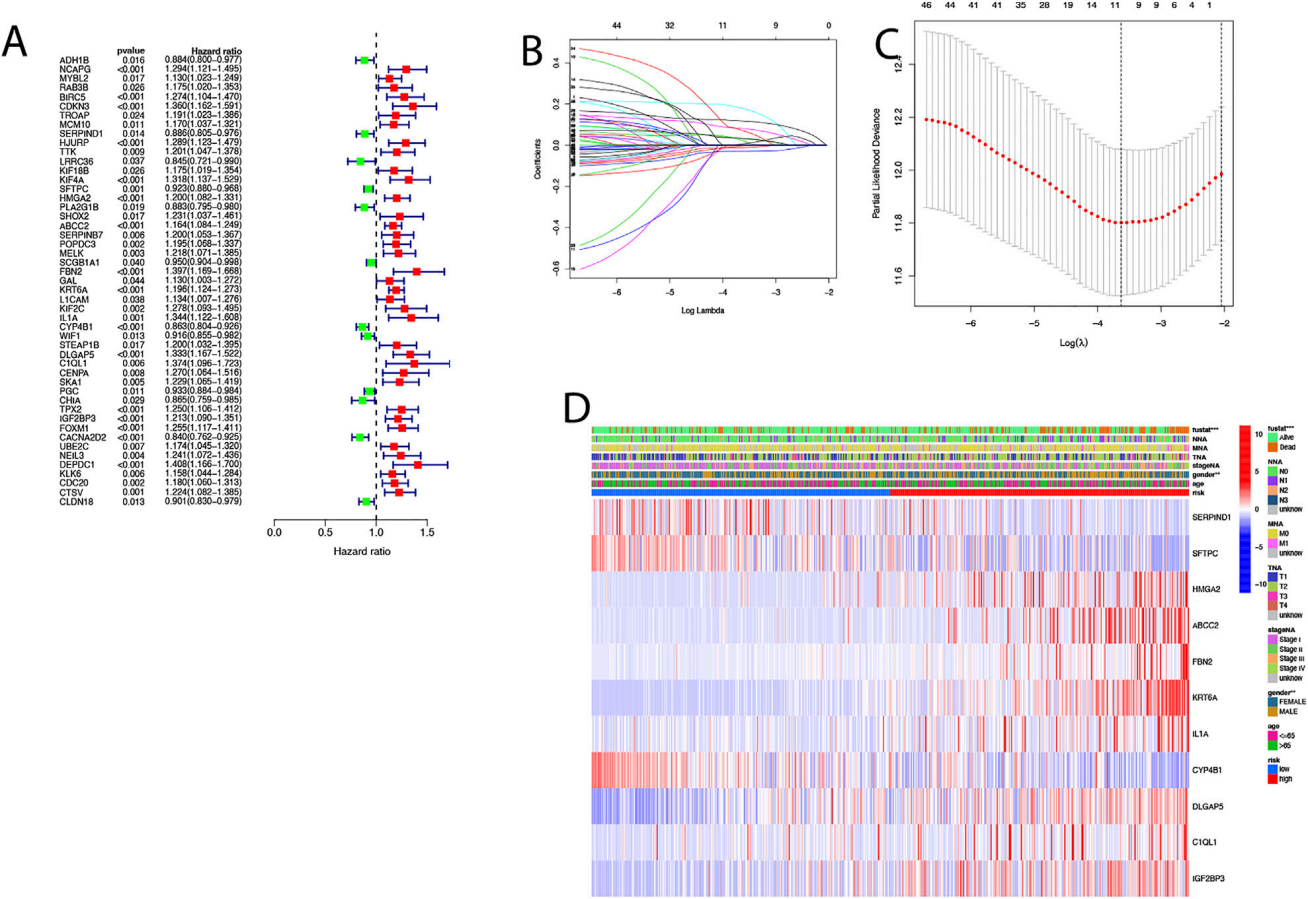
Construction of risk signature in the TCGA cohort. **(A)** Univariate cox regression analysis of LUAD for each apoptosis-related gene, *p* < 0.05. **(B)** In the LASSO-Cox model of the TCGA cohort, the minimum standard was adopted to obtain the value of the super parameter λ by 10-fold cross-validation. **(C)** Cross-validation for tuning the parameter selection in the LASSO regression. **(D)** Heat map showing the clinical characteristics of 11 model genes.

In order to develop a model that could quantify the ideal prognosis for each patient, we took a sample of 494 LUAD cases with complete survival information for the study. We firstly identified 153 differentially expressed genes between the two clusters and then used univariate Cox regression to analyze these 153 differentially expressed genes for initial screening (Figure 5A; Supplementary Table S5). The LASSO-Cox regression model was applied to include 11 of the differential genes with a minimum value of λ(Figures 5B,C). An apoptosis-related signature score was established which we named “AS-score”; a genetic risk score was constructed based on the optimal *λ*-value and calculated as: AS-score = (−0.003*SERPIND1 exp.) + (−0.118*SFTPC exp.) + (0.033*HMGA2 exp.) + (−0.081*ABCC2 exp.) + (0.178*FBN2 exp.) + (0.117*KRT6A exp.) + (0.022*IL1A exp.) + (−0.030*CYP4B1 exp.) + (0.007*DLGAP5 exp.) + (0.071*C1QL1 exp.) + (0.033*IGF2BP3exp.). The heatmap showed the relationship between 11 model genes and clinical characteristics (including stage TMN staging, gender, survival status, etc.) (Figure 5D).

**FIGURE 6:**
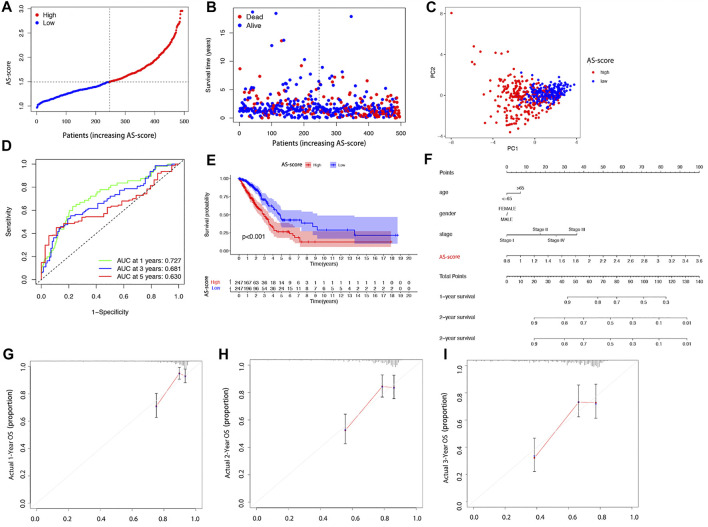
Overall performance of the 11 gene signatures in all cohorts **(A)** Patient distribution based on risk scores. **(B)** Survival status of each patient (low-risk group: left side of the dashed line; high-risk group: right side of dashed line). **(C)** PCA plots of LUAD patients based on risk scores. **(D)** The ROC curve shows the predictive efficiency of the risk score. **(E)** Kaplan-Meier curves for LUAD patients in high and low-risk groups. **(F)** Nomogram for predicting 1, 2, and 3 years overall survival for LUAD patients in TCGA cohort. **(G–I)** Calibration plots of predicted recurrence after 1, 2, and 3 years. The x-axis represents the predicted probability of recurrence in the nomogram, and the y-axis represents the actual probability of recurrence.

The 494 LUAD patients were equally divided into high AS-score and low AS-score groups based on the median score calculated by the risk score calculation formula (Figures 6A,B). Principal component analysis (PCA) showed that patients with different risks could be divided into two groups (Figure 6C). The sensitivity and specificity of the prognostic model were assessed using time-dependent receiver operating characteristic curve (ROC) analysis. We found that the area under the ROC curve (AUC) was 0.727 at 1 year, 0.681 at 2 years, and 0.630 at 3 years (Figure 6D). Kaplan-Meier analysis suggested a significant difference in OS between the high AS-score group and the low AS-score group (*p* < 0.001), with the high AS-score group having a lower survival time than the low AS-score group (Figure 6E). To create a quantitative tool that can predict the clinical application of OS in LUAD patients, we developed a nomogram for predicting 1-, 2- and 3-years s overall survival for LUAD patients in the TCGA cohort (Figure 6F). Calibration plots showed that the nomogram agrees with the ideal model in the TCGA cohort (Figures 6G–I).

### External Validation of the Risk Signature

**FIGURE 7:**
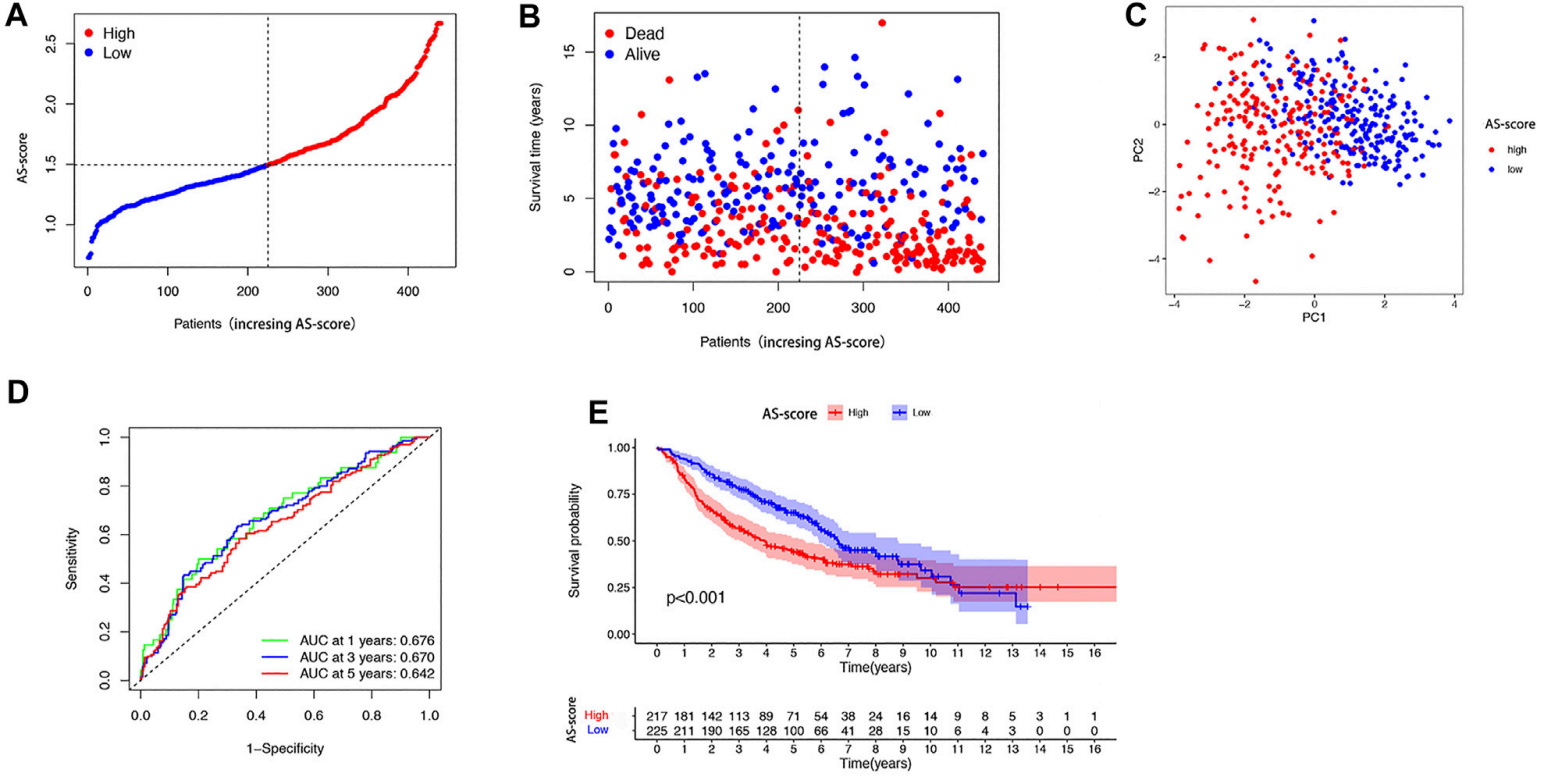
Validation of risk prediction models in the GEO cohort **(A)** Distribution of patients based on risk score in the GEO cohort. **(B)** Survival status of each patient in the GEO cohort based on the risk prediction model (low-risk group: left side of the dashed line; high-risk group: right side of the dashed line). **(C)** PCA mapping of LUAD patients in the GEO cohort based on risk scores. **(D)** ROC curve showing the predictive efficiency of risk scores in the GEO cohort. **(E)** Kaplan-Meier curves for LUAD patients in high and low-risk groups.

The GSE68465 cohort of 443 LUAD patients with survival information was used as the validation set. According to the AS-score median risk score, 225 patients in the GSE68465 cohort were divided into low AS-score group and 217 into high AS-score group (Figures 7A,B). Principal component analysis (PCA) showed that patients could be well classified into high and low groups based on the AS-score (Figure 7C). ROC curve analysis of the GEO cohort showed that the constructed model was a good predictor (1-year AUC = 0.676, 2-years AUC = 0.670, 3-years survival 0.642) (Figure 7D). Kaplan-Meier analysis also showed a significant difference in survival between the low AS-score and high AS-score groups (*p* < 0.001). The high AS-score group had a significantly lower survival time than the low AS-score group (Figure 7E).

### Independent Prognostic Value and Functional Analysis of Risk Signature

**FIGURE 8:**
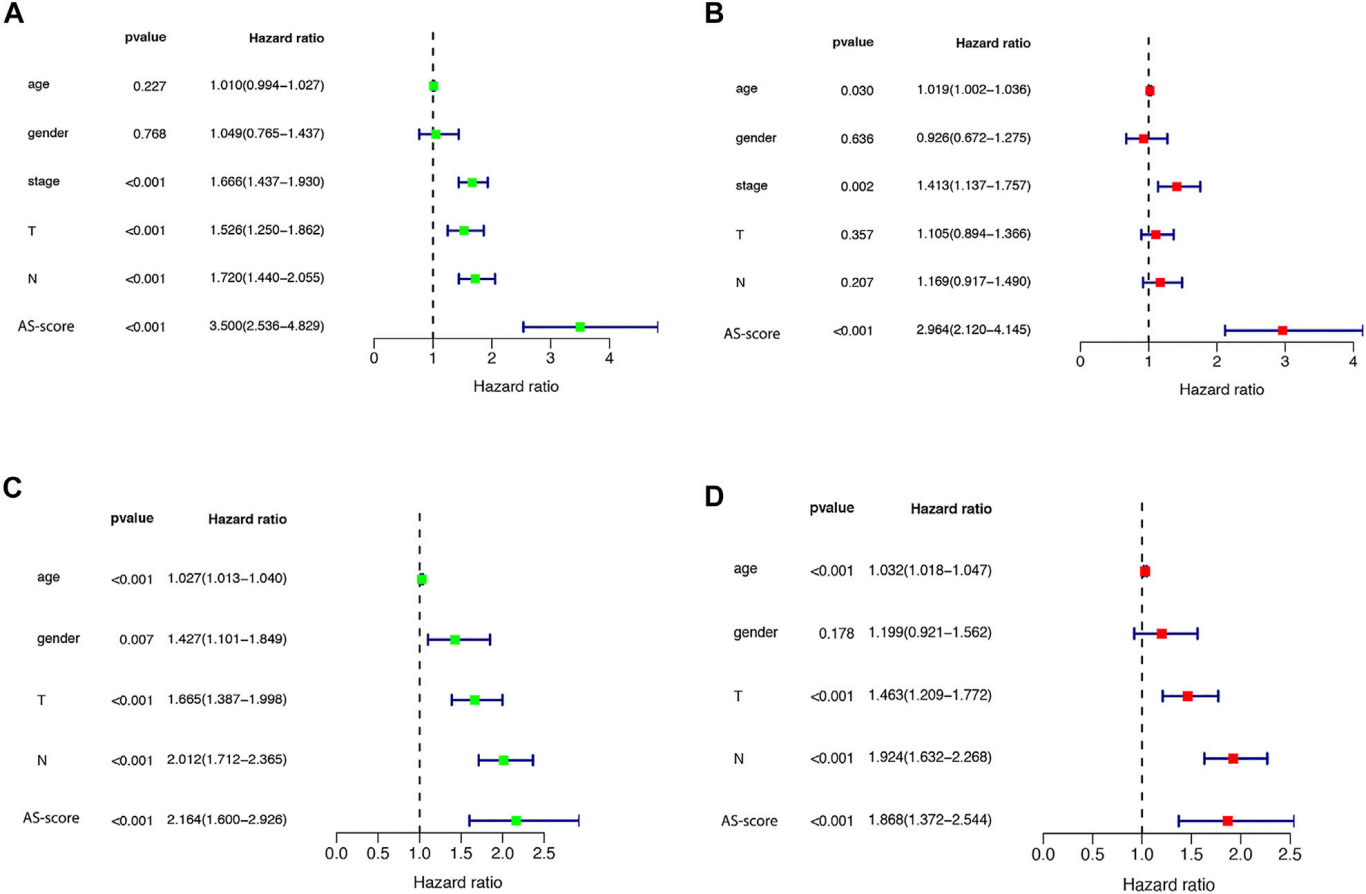
Univariate and multivariate Cox regression analysis of risk scores **(A)** Univariate cox regression analysis in the TCGA cohort. **(B)** Multivariate Cox regression analysis in the TCGA cohort. **(C)** Univariate cox regression analysis in the GEO (GSE68465) cohort. **(D)** Multivariate Cox regression analysis in the GEO (GSE68465) cohort.

Univariate and multivariate Cox regression analyses were used to assess whether the risk score from the genetic trait model could be used as an independent prognostic factor. Univariate Cox regression analysis showed that period, T-stage, lymph node metastasis (N), and risk score were all factors associated with prognosis in the TCGA cohort and in the GSE68465 cohort (HR = 3.500, 95% CI: 2.536–4.829 and HR: 2.163, 95% CI: 1.600–2.926) (Figures 8A,B). Multivariate Cox regression analysis suggested that risk score is an independent prognostic factor after adjusting for other confounders (HR = 2.964, 95% CI: 2.120–4.145 and HR: 1.868, 95% CI: 1.372–2.544) (Figures 8C,D).

### Comparison of Immunoreactivity

**FIGURE 9:**
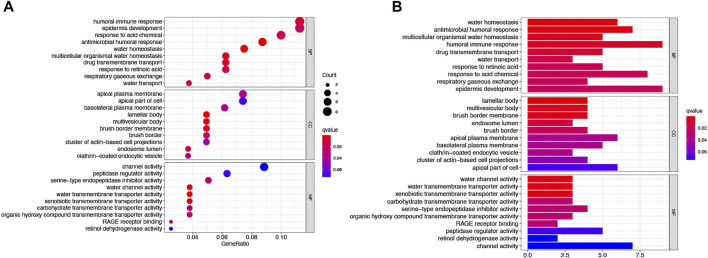
Functional analysis of the DEGs between two risk groups in the TCGA cohort. **(A)** Bubble plot graph showing GO enrichment of DEGs between the two groups in the TCGA cohort (longer bars indicate more gene enrichment, and darker red indicates more pronounced differences q-value: the adjusted *p*-value). **(B)** Barplot showing the KEGG pathway of DEGs between the two groups in the TCGA cohort (longer bars indicate greater gene enrichment, and darker red indicates more pronounced differences).

To further explore differences in gene function and pathways between risk model classifications, we identified differential genes between low AS-scores and high AS-scores in the TCGA cohort. GO enrichment analysis based on these DEGs showed that the DEGs were mainly associated with signaling pathways for the immune response, epidermal development (Figures 9A,B).

**FIGURE 10:**
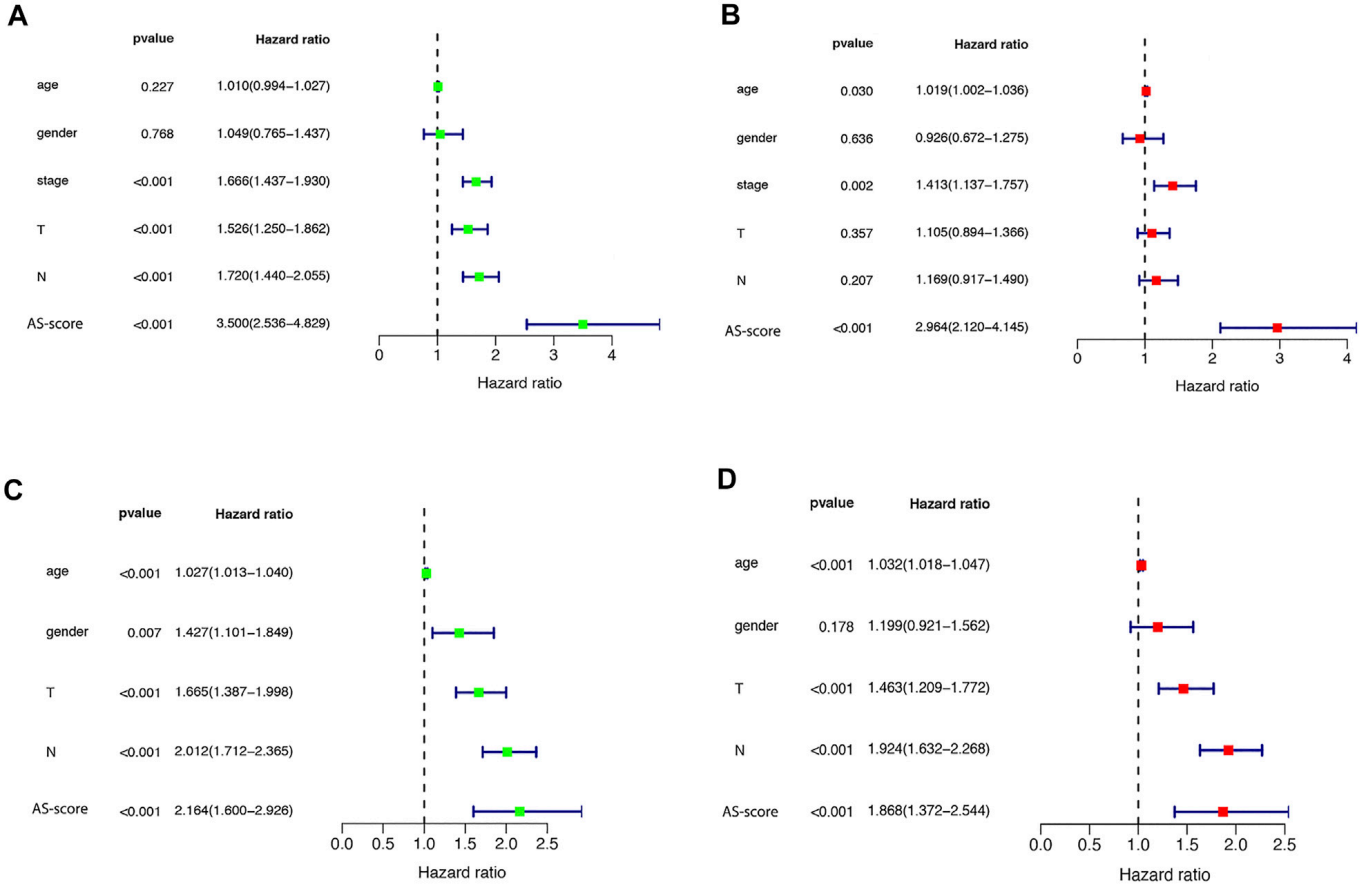
Comparison of ssGSEA scores between the two risk groups **(A)** Immune cell infiltration between different risk groups in the TCGA cohort. **(B)** Immune-related functions between the two risk groups in the TCGA cohort. **(C)** Immune cell infiltration between different risk groups in the GEO (GSE68465) cohort. **(D)** Immune-related functions between the two risk groups in the GEO (GSE68465) cohort.

Increasing studies have shown that tumor metastasis and invasion are inseparable from the tumor microenvironment. Based on the functional analysis, we further explored the role of the constructed AS-score in the immune microenvironment and immunotherapy. Single sample gene set enrichment analysis (ss GSEA) was used to compare the enrichment scores of 16 immune cell types and the activity of 13 immune-related pathways in the TCGA and GSE6846 cohorts in low AS-score versus high AS-score populations. In the TCGA cohort, the high AS-score group had lower levels of immune cell infiltration relative to the low AS-score group, except for p DCs Th1-cells, especially a DCs and I DCs cells. In contrast, in the immune pathway, the high AS-score was mainly enriched in HLA, MHC_class_1, Type_II_IFN_response (Figures 10A,B). Similar conclusions were reached in the study of immune infiltration in the GSE6846 cohort (Figures 10C,D).

**FIGURE 11:**
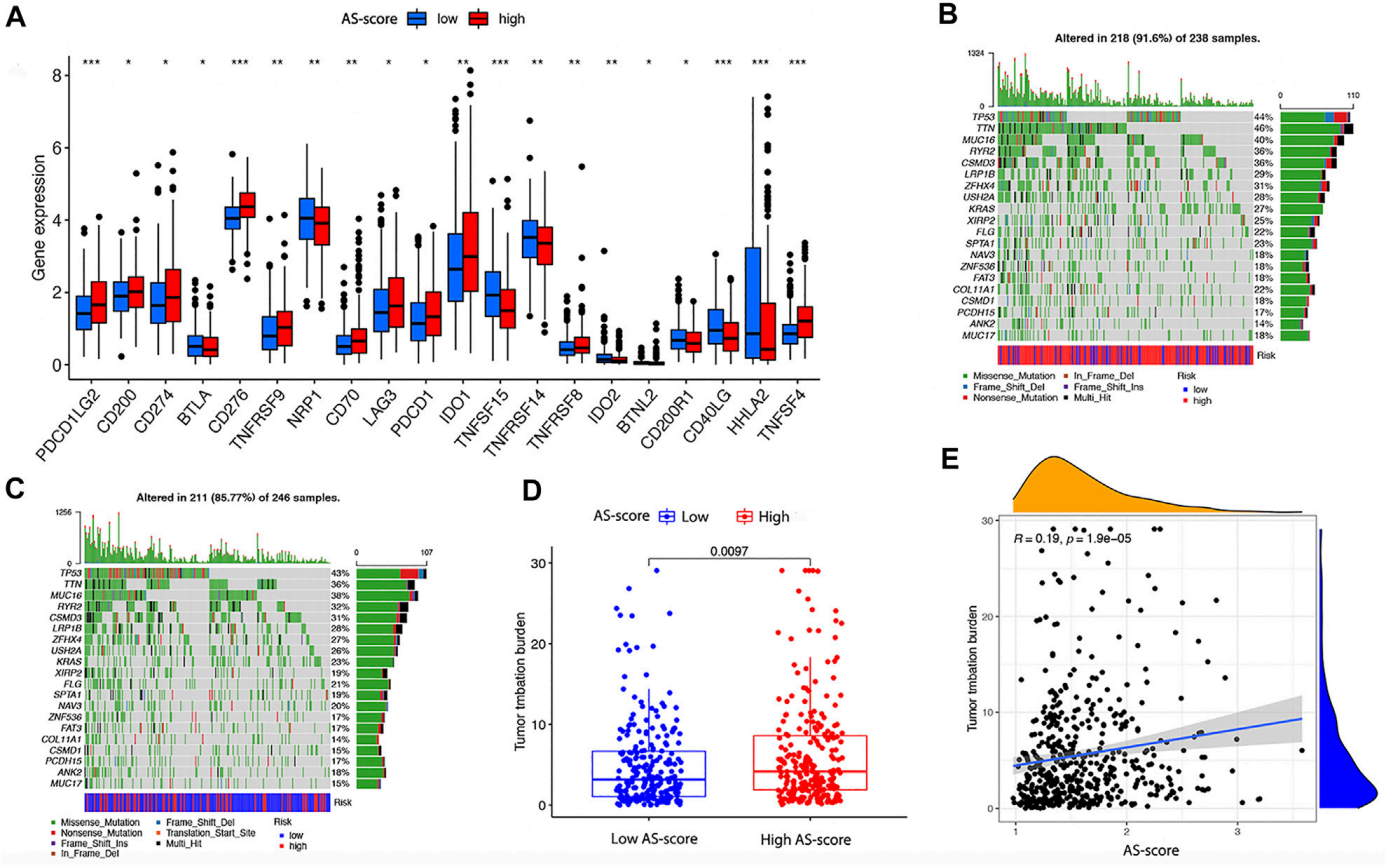
Differences in immune checkpoint and TMB between the two risk groups in the TCGA cohort **(A)** Differences in immune checkpoints between the two risk groups in the TCGA cohort. **(B)** TMB in the high AS-score group in the TCGA cohort. **(C)** TMB in the low AS-score group in the TCGA cohort. **(D)** Differences of TMB between high and low AS-score groups in the TCGA cohort. **(E)** Association between AS-score and TMB in the TCGA cohort.

Large studies have revealed that immunotherapy is emerging as a new hope for cancer treatment, and immune checkpoints play an important role in the immune response (Marin-Acevedo et al., 2018). To further explore the impact of high and low AS-score groups on immunotherapy, we compared the differences between immune checkpoints between the two groups. As we found, the expression of PDCDLG2, CD274, TNFSF15, CD40LG, HHLA2 was significantly upregulated, whereas CD276 and TNFSF4 were downregulated considerably in the high AS-score group, suggesting that apoptosis-related characteristics scored higher in patients who may have a better chance to immunotherapy (Figure 11A). Current studies suggested that patients with high TMB show more significant benefits from PD-1/PD-L1 inhibition than patients with low TMB (Hodges et al., 2017; Rizvi et al., 2018).

To further compare the mutations profile between the two AS-score groups, we examined mutation information for high and low AS-scores separately. In the TCGA cohort, 218 (91.6%) of the 238 samples in the high AS-score group had mutations, with the highest mutation frequency being in TTN (Figure 11B).In contrast, 211 of 246 samples (85.77%) in the low AS-score group showed mutations, with the most frequent mutation being TP53 (Figure 11C). At the genetic level, the TMB was higher in the high AS-score group compared to the low AS-score group (*p* = 0.0097) (Figure 11D). Furthermore, the risk index was positively correlated with the TMB, with an increased AS-score (*p* = 1.9e-05) (Figure 11E). This study indirectly suggested that AS- score plays a key role in mediating the clinical responses to checkpoint immunotherapy.

### The Role of the AS-Score in Antineoplastic Drug Therapy

**FIGURE 12:**
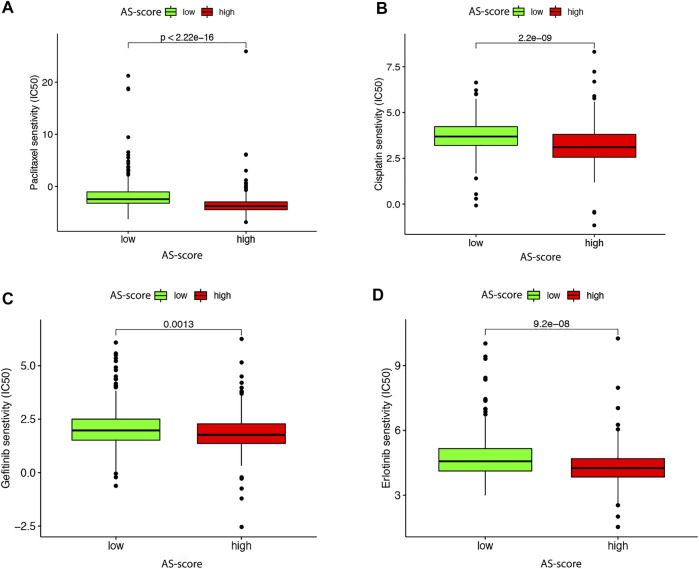
Differences in antitumor drug therapeutic efficacy between high and low AS-scores in the TCGA cohort **(A)** Differences in paclitaxel therapeutic efficacy between high and low AS-score in the TCGA cohort. **(B)** Differences in the efficacy of cisplatin therapeutics between high and low AS-score in the TCGA cohort. **(C)** Differences in gefitinib therapeutic efficacy between high and low AS-score in the TCGA cohort. **(D)** Differences in the therapeutic efficacy of erlotinib between high and low AS-score in the TCGA cohort.

It has been shown that TMB predicts the efficacy of immunotherapy and influences the response to chemotherapy and targeted therapies. Therefore, we investigated the association between this AS-score and the effectiveness of chemotherapy and targeted therapies in LUAD patients (Choucair et al., 2020). To compare the efficacy of the high and low AS-score groups to commonly used chemotherapeutic and targeted drugs. The results showed that the high AS-score group had a lower half-maximal inhibitory concentration (IC50) of cisplatin (*p* = 2.2e-09) and paclitaxel (*p* < 2.22e-16) compared to the low AS-score group (Figures 12A,B), suggesting a higher sensitivity to treatment. Similarly, the high AS-score group had lower IC50 for erlotinib (*p* = 9.2e-08) and gefitinib (*p* = 0.0013) (Figures 12C,D), suggesting a better sensitivity to treatment with targeted drugs as well. These results suggested that the AS-score can predict the effect of treatment with chemotherapeutic drugs and targeted drugs.

### Validation in LUAD Cell Lines

**FIGURE 13:**
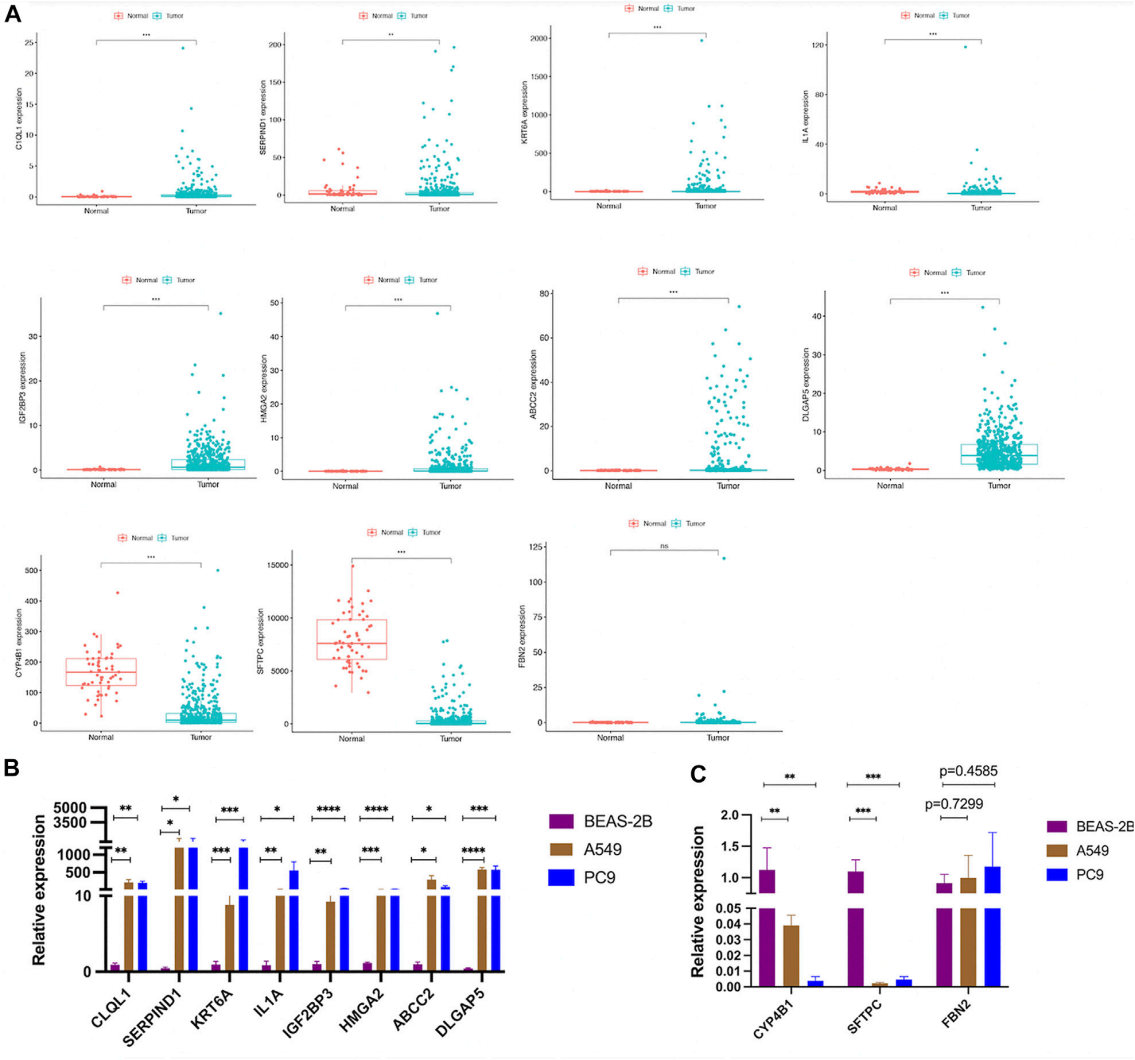
Validation of the differential expression of the 11 model genes **(A)** Expression of 11 model genes from the TCGA cohort in LUAD tissue. **(B,C)** Validation of differential expression of the 11 model genes in LUAD cell lines.

To better analyze the AS-score signatures of these 11 gene constructs, we predicted their expression in the TCGA cohort. The results showed that CYP4B1 and SFTPC were lowly expressed in LUAD, SERPIND1, HMGA2, ABCC2, KRT6A, IL1A, DLGAP5, C1QL1, IGF2BP3 were highly expressed, while FBN2 was not differentially expressed between LUAD and normal tissues (Figure 13A). Subsequently, we validated the expression of the 11 genes incorporated into the model constructs in the cell lines following the steps described above and found general agreement with the predicted results by comparing the differential expression of each gene in normal bronchial epithelial cells (BEAS-2B) versus A549 and PC9 (Figures 13B,C).

## Discussion

As the most common type of LC, LUAD is currently treated by surgery, radiotherapy, targeted therapy, and immunotherapy (Siegel et al., 2021).

Apoptosis, as a form of programmed cell death (PCD), is mediated through multiple signaling pathways (classified mainly as intrinsic and extrinsic pathways) (Pistritto et al., 2016). The intrinsic apoptotic pathway (mitochondrial-dependent) is mediated by intracellular signals that converge at the mitochondrial level in response to different stress conditions, with internal stimuli such as irreparable genetic damage, hypoxia, extremely high cytoplasmic Ca + concentrations, and severe oxidative stress being important factors in initiating the intrinsic mitochondrial pathway (Green and Kroemer, 2004; Riley et al., 2018). In contrast, the extrinsic apoptotic pathway (death receptor-dependent) is initiated by the interaction of death receptors of the tumor necrosis factor receptor (TNFR) superfamily exposed on the cell surface with the respective protein TNF family ligands (Guicciardi and Gores, 2009). The apoptotic process sequentially and efficiently removes cells that cause damage (e.g., DNA damage or cells generated during development), thereby maintaining cell renewal, embryonic development, and immune system activity. Interaction of apoptotic pathways with other signaling mechanisms also affects cell death (Bauer and Helfand, 2006). Dysregulation of apoptotic cell death mechanisms is a feature of cancer, as shown by a growing body of literature (Lauber and Herrmann, 2015). The altered apoptosis is not only associated with tumor development and progression but also with resistance to antitumor drugs, and therapeutic strategies targeting apoptosis-resistant molecules are an effective way to restore the sensitivity of cancer cells to apoptosis and enhance antitumor effect (Kim, 2005; Mohammad et al., 2015).

However, it is unclear how apoptosis-associated genes interact in LUAD and whether these genes are associated with clinical characteristics of patients, prediction of antitumor drug efficacy, and infiltration in the immunological microenvironment. Classification of samples based on predefined gene expression profiles is a proven method (Cristescu et al., 2015). In this study, we used this method to analyze the data information of TCGA-LUAD. The 136 genes associated with apoptosis were investigated between LUAD and normal tissues, and the results revealed differences in the expression of most of these genes. Protein-protein interaction networks were mapped to demonstrate the interrelationships. According to the DEARGs associated with apoptosis, two distinct subtypes existed by consensus clustering analysis. There were significant differences in survival and prognosis between the two subtypes. To further assess the prognostic value of these associated regulators, we initially performed a preliminary screening of these differential genes using univariate analysis and then constructed an 11 (SERPIND1, SFTPC, HMGAA2, ABCC2, FBN2, KRT6A, IL-1A, CYP4B1, DLGAP5 C1QL1, IGF2BP3) genetic risk score model, named AS-score. LUAD patients in the TCGA cohort were then divided into high and low AS-score groups based on the median AS-score score. The performance from this risk model score was validated in the GSE6846 cohort. Both the TCGA and GSE6846 cohorts showed that the low AS-score group had better prognostic survival and prognostic performance than the high AS-score group. On the basis that this AS-score is a good predictor of patient prognosis and an independent prognostic risk factor for LUAD, we validated its expression in LUAD cell lines versus normal bronchial epithelial cells. We found it to be consistent with database predictions.

It has been shown that the tumor microenvironment is significantly related to the clinical features, genomic expression, and biological characteristics of tumor patients (Zhang et al., 2020). A comprehensive analysis of the role of the tumor microenvironment in LUAD will help clarify the tumor immunophenotype of LUAD, explore independent prognostic indicators and new therapeutic targets, thus improving patient prognosis and predicting the effectiveness of immunotherapy (Liotta and Kohn, 2001; Fang and Declerck, 2013). Previous studies have also shown that the tumor microenvironment plays a key role in tumor carcinogenesis and revealed significant epigenetic regulators, opening new avenues for precision and personalized medicine (Gadiyar et al., 2020). According to our findings, differentially expressed genes between the low AS-score group and the high AS-score group were associated with immune-related pathways. Comparison of immune cell infiltration and activation pathways between the low AS-score and high AS-score groups showed differences in multiple cellular and immune pathways.

Current immunotherapy for LUAD is mainly directed at immune checkpoint inhibitors (Marin-Acevedo et al., 2018; Wang et al., 2020). The most common and rapidly developing of these are PD-L1 and PD-1 inhibitors, which exert antitumor effects by blocking the binding of PD-L1 to PD-1, thereby reducing the inhibition of T-cell activation, suggesting that high PD-L1 expression is more effective for immunosuppressive therapy (Zhu et al., 2018; Wang et al., 2021).

In order to further explore the differential impacts on immunity between the high and low AS-score, we compared the TMB. We found that the high AS-score group had more mutational load than the low AS-score group and that AS-score was positively correlated with TMB. Rizvi et al. (2018) suggested that TMB and PD-L1 expression, although not correlated, independently predicted the efficacy of immunosuppressive therapy and combined TMB and PD-L1 expression into a multivariate prediction model should yield greater predictive capability. We also showed that the high AS-score group had higher PD-L1expression but TMB than the low AS-score group, and there may be a consistent synergistic predictive effect between the two. The high AS-score group had better immunotherapy efficacy than the low AS-score group (Samstein et al., 2019).

The prediction model based on DEARGs showed that the high AS-score group achieved better efficacy in immunotherapy. Similarly, the high AS-score group achieved better efficacy with chemotherapy and targeted drug therapy. At the same time, Ludovic Fournel (Fournel et al., 2019) found that cisplatin-based induction chemotherapy increased PD-L1 expression in tumor cells, suggesting that chemotherapy combined with immunotherapy could improve the overall prognosis of patients with LUAD. And the combination of cisplatin and anti-PD - L1 therapy improved the response to tumor treatment, which was consistent with the predictions of the AS-score model. The results of this study showed that although the low AS-score group had better survival than the high AS-score group. But fortunately, the high AS-score group was more sensitive to chemotherapy and targeted therapy. Meanwhile, because cisplatin-based therapy could increase the expression of PD-L1 in tumor cells, suggesting that the high AS-score group is more sensitive to chemotherapy combined with targeted therapy. Therefore, it is reasonable to assume that this model may indicate antitumor therapy.

In conclusion, our study showed that this AS-score plays a role in clinical prognosis and sensitivity to antitumor drug therapy. Our study provides a new genetic marker for predicting the prognosis of patients with LUAD and provides an important basis for further research into the relationship and new apoptosis-related antitumor drug treatments.

## Data Availability

The data could be downloaded at (https://portal.gdc.cancer.gov/, https://xenabrowser.net/, and https://www.ncbi.nlm.nih.gov/geo/; GSE68465) and the code used during the current study are available from the corresponding author on reasonable request.

## Abbreviations

DEARGs, differentially expressed apoptosis-related gene; DEGs, differentially expressed genes; GDSC, genomics of drug sensitivity in cancer; GO, gene ontology; IC50, half-maximal inhibitory concentration; KEGG, kyoto encyclopedia of genes and genomes; LASSO, least absolute shrinkage and selection operator; LC, lung cancer; LUAD, lung adenocarcinoma; NSCLC, non-small cell lung cancer; PCA, principal component analysis; ROC, receiver operating characteristic curve; SCLC, small cell lung cancer; ss GSEA, single sample gene set enrichment analysis; STRING, search tool for the retrieval of interacting genes; TCGA, the cancer genome atlas; TMB, tumor mutational burden.
